# Characterization and Differentiation of *Mycobacterium avium* subsp. *paratuberculosis* from Other Mycobacteria Using Matrix Assisted Laser Desorption/Ionization Time-of-Flight Mass Spectrometry

**DOI:** 10.3389/fcimb.2017.00297

**Published:** 2017-06-30

**Authors:** Subbarao V. Ravva, Leslie A. Harden, Chester Z. Sarreal

**Affiliations:** Produce Safety and Microbiology Research Unit, Western Regional Research Center, Agricultural Research Service, United States Department of AgricultureAlbany, CA, United States

**Keywords:** MALDI-TOF, Mycobacterium, *Mycobacterium avium avium*, *Mycobacterium avium paratuberculosis*, mass spectrometry

## Abstract

*Mycobacterium avium* subsp. *paratuberculosis* (MAP), the causative agent of Johne's disease in cattle, is responsible for significant economic losses to the US dairy industry. The pathogen has also been associated with chronic human diseases like Crohn's disease, type 1 diabetes and multiple sclerosis. Determining causation requires rapid characterization and source tracking the pathogen. Here, we used matrix-assisted laser desorption/ionization time-of-flight (MALDI-TOF) mass spectrometry to characterize and differentiate strains of MAP from 14 other species of *Mycobacterium* from bovine, human, and environmental sources. Lysates from cells disrupted by bead beating in TFA-acetonitrile solution were analyzed by MALDI-TOF. MAP strains were differentiated by mass spectral profiles that are distinct from each other and from other *Mycobacterium* species. Cluster analysis of spectral profiles indicates two distinct clusters, one dominated by the members of avium complex and a second group dominated by members of *fortuitum* and *parafortuitum* complexes. We believe that MALDI-TOF methods can be used to differentiate and source-track MAP strains.

## Introduction

*Mycobacterium avium* subsp. *paratuberculosis* (MAP), the causative agent of Johne's disease in cattle, is responsible for an annual loss of 200–250 million dollars to the US dairy industry (Ott et al., 1999). Johne's disease is a debilitating chronic infectious enteritis of ruminants whose spread can only be controlled by culling. There is no cure. Herd level prevalence was estimated to be as high as 91% (Lombard et al., 2013) and spread of the pathogen is through the fecal-oral route and also through milk. Early pathogen detection coupled with culling and introduction of MAP-free animals into the herd is the only option for pathogen-free dairies.

MAP has been associated with different autoimmune diseases such as Crohn's disease (Sechi et al., 2005; Chiodini et al., 2012), type 1 diabetes (Sechi et al., 2008; Cossu et al., 2011; Masala et al., 2011), and multiple sclerosis (Cossu et al., 2013; Frau et al., 2013) but casual links were not established. Clinical manifestations of Crohn's disease partly resemble the clinical signs of Johne's disease in ruminants (Overduin et al., 2004). Milk contaminated with MAP has been considered to be a potential source of exposure to humans (Grant, 2005). Both live and dead cells of MAP were detected in pasteurized milk (Millar et al., 1996; Grant et al., 2002). Determining causation requires sensitive methods to detect and isolate the pathogen and methods for source tracking.

Isolation of the pathogen using traditional culture-based methods can take up to 20 weeks (Whittington, 2009). Thus, real-time quantitative PCR methods based on the detection of insertion sequence targets IS900 and ISMav2 were developed for rapid and sensitive detection of MAP (Ravva and Stanker, 2005; Sting et al., 2014). PCR methods can be coupled with magnetic-bead separations for cleanup and concentration of MAP DNA from complex fecal samples (Leite et al., 2013; Sting et al., 2014). PCR methods can rapidly detect the presence of MAP, but they cannot aid in typing the strains necessary for source tracking the pathogen.

Molecular typing methods were developed that discriminate strains of MAP. IS900 restriction fragment length polymorphism (RFLP) typing and multilocus variable-number tandem-repeat analysis (MLVA) methods were used to type MAP strains from humans and cattle (Overduin et al., 2004). In one study, the human isolates were found to be genetically indistinguishable from the cattle strains and the authors suggested that humans can be infected with strains from cattle. However, this method lacked resolution in discriminating strains as 82% of strains tested were of one MLVA type. Other studies that used tandem application of mycobacterial interspersed repetitive unit (MIRU) and multilocus short sequence repeat (MLSSR) genotyping methods were able to distinguish sheep isolates from cattle isolates (Amonsin et al., 2004) and generated 22 distinct genotypes from 38 MAP strains (Douarre et al., 2011).

Matrix-assisted laser desorption ionization time-of-flight (MALDI-TOF) mass spectrometry (MS) detection of biomolecules in cell-free extracts or culture supernatants have been used to characterize and discriminate strains of *Escherichia coli* O157:H7 and *Campylobacter* (Fagerquist et al., 2005, 2010; Mandrell et al., 2005). MALDI-TOF MS has been recently used to characterize mycobacteria (Park et al., 2016; Samli and Ilki, 2016; Zingue et al., 2016). However, much of the work was focused on differentiating pathogenic strains of *Mycobacterium tuberculosis* from other species of mycobacteria including some members of the *M. avium* complex. Although, MAP was included in a couple of studies (Pignone et al., 2006; El Khechine et al., 2011), mass spectral data was either not published or spectral data was limited to a mass range of <5,000 Da (Pignone et al., 2006). These studies also lacked uniformity in sample preparation. Samples for mass spectrometry included whole cells in matrix solution (Lin et al., 2013), cells directly applied on MALDI plates (Pignone et al., 2006), non-sonicated cell-free supernatants (Hettick et al., 2006), mechanical breaking of heat-inactivated cells followed by protein extractions with formic acid and acetonitrile (El Khechine et al., 2011) or protein extraction from culture supernatants (Marekovic et al., 2016). Cell lysates were used routinely for MALDI-TOF characterization of foodborne pathogens in our laboratory (Mandrell et al., 2005; Fagerquist et al., 2010; Penny et al., 2016).

We predicted that MALDI-TOF characterization of biomolecules in cell lysates would provide valuable data for characterization and differentiation of MAP from other mycobacteria and for typing MAP strains. Thus, MAP strains and other mycobacteria from different host species and environments were characterized by MALDI-TOF MS. Spectral data was analyzed using Bionumerics software to obtain similarities in spectral patterns and unique finger prints that differentiate *Mycobacterium* species and strains of MAP.

## Materials and methods

### Mycobacterial cultures

Six different strains of MAP and 14 different *Mycobacterium* species from different host species and environments (Table 1) were evaluated. Mycobacterial cells were grown in 50 mL of 7H9 broth supplemented with 10% (vol/vol) Middlebrook OADC growth supplement (Becton Dickinson, Franklin Lakes, NJ), 0.5% Tween 80 and 0.0002% (wt/vol) mycobactin J (Allied Monitor Inc., Fayette, MO) (Chang et al., 2002). The growth medium was supplemented with 50 μg/mL of amphotericin B and 100 μg/mL each of nalidixic acid and vancomycin to reduce contaminant growth during the 2–4 months of incubation required for MAP (Collins et al., 1990). The cultures were grown at 37°C on a gyratory shaker at 200 rpm. Mycobacteria other than MAP strains were harvested within 1–2 weeks of incubation.

**Table 1:** Mycobacterial strains used.

**Mycobacterium species**	**Complex or clade**	**Runyon group**	**Strain**	**Source**
MAP	*M. avium* complex	III	ATCC 19698^a^	Bovine
MAP	*M. avium* complex	III	NADC 1038^b^	Bovine
MAP	*M. avium* complex	III	ATCC 43015	Human
MAP	*M. avium* complex	III	NADC Kay	Bovine
MAP	*M. avium* complex	III	NADC 1040	Bovine
MAP	*M. avium* complex	III	NADC 5016	Bovine
*M. avium* subsp. *avium*	*M. avium* complex	III	ATCC 25291	Chicken
*M. avium* subsp. *Silvaticum*	*M. avium* complex	III	ATCC 49884	Wood pigeon
*M. nonchromogenicum*	*M. nonchromogenicum/terrae* complex	III	ATCC 19530	Soil
*M. fortuitum* subsp. *Fortuitum*	*M. fortuitum* clade	IV	ATCC 6841	Human
*M. senegalense*	*M. fortuitum* clade	IV	ATCC 35796	Bovine
*M. porcinum*	*M. fortuitum* clade	IV	ATCC 33776	Swine
*M. vaccae*	*M. vaccae* clade	IV	ATCC 15483	Bovine
*M. austroafricanum*	*M. vaccae* clade	IV	ATCC 33464	Soil
*M. neoaurum*	*M. neoaurum* clade	IV	ATCC 25795	Soil
*M. diernhoferi*	*M. neoaurum* clade	IV	ATCC 19340	Dairy water trough
*M. farcinogenes*	*M. fortuitum* clade	IV	ATCC 35753	Bovine
*M. thermoresistibile*	*M. smegmatis* clade	IV	ATCC 19527	Soil
*M. smegmatis*	*M. smegmatis* clade	IV	ATCC 19420	Human
*M. scrofulaceum*	*M. scrofulaceum complex*	II	ATCC 19981	Human

a *ATCC-American Type Culture Collection, Manassas, VA*.

b *NADC-National Animal Disease Center, Ames, IA*.

### Cell lysates for MALDI-TOF MS

Protein extraction solution was prepared by combining 1:2 acetonitrile-water with 0.1% sequencing-grade trifluoroacetic acid (TFA) (Sigma-Aldrich, St. Louis, MO).

Cultured cells (0.1 mL) were centrifuged at 18,000 × g for 5 min. Pelleted cells were bead-beat (Mini-Beadbeater-16; BioSpec Products Inc., Bartlesville, OK) 3 cycles in 0.3 ml of TFA extraction solution containing ~75 mg of 0.1-mm zirconia-silica beads (BioSpec). Each cycle consisted of 1 min of bead-beating followed by 2 min of cooling on ice. After bead-beat treatment, TFA extracted cell slurry was centrifuged again at 18,000 × g for 5 min and then 0.2 mL supernatant was removed for analysis. OD_280_ measurements were taken using a 1:6 ratio of lysate to TFA extraction solution. Measurements ranged from 0.24 to 0.70 with an average value of 0.31, and normalized to 0.23 for mass spectral runs.

### MALDI-TOF sample preparation and operation

A saturated solution of *trans*-4-hydroxy-3-methoxy-cinammic acid (ferulic acid) (Sigma-Aldrich, St. Louis, MO) was prepared in 0.5 mL of the above TFA extraction solution. A working matrix solution was prepared by diluting 200 μL of saturated solution with 100 μL of the TFA extraction solution. The matrix solution was briefly vortexed and 0.5 μL was deposited at room temperature for each sample spot and allowed to air dry on a 7 by 7 spot homemade stainless steel MALDI target plate. For each sample of cell lysate supernatant, 0.5 μL was deposited onto a dried matrix spot and samples allowed to air dry at room temperature. All experiments were run in the positive ion mode on a Bruker Daltonics (Billerica, MA) Reflex II MALDI-TOF mass spectrometer operated in reflectron mode with delayed extraction; the delayed extraction setting was set to “medium.” Sample ionization was achieved with a nitrogen laser at 337 nm. A minimum of 200 laser shots per sample was used to generate each spectrum. The instrument was externally calibrated using the adrenocorticotropic hormone clip (peptide 18–39), angiotensin II, substance P, bovine insulin, and horse heart myoglobin as molecular weight standards (Mandrell et al., 2005). In addition, cell lysates from MAP type strain ATCC 19698 were included along with samples run on different days to determine the reproducibility of spectral profiles. Data was processed using the Bruker Flex Analysis software.

### Spectral analysis

Raw spectral data was exported as text files and analyzed using the fingerprint data module of Bionumerics v7.1 (Applied Maths, Sint-Martens-Latem, Belgium). Spectral comparisons were made using the automated workflow with standard baseline correction. Signal-to-noise ratio corrections were made for each spectrum with a value between 35 and 55 to eliminate background noise from the peaks. The noise reduction in spectral peaks did not eliminate any important banding information. Band matching analysis was used to determine peak band patterns. Spectral characterization was targeted for mass ions with mass to charge ratios of 3–11 kDa. By judicious baseline correction and noise reduction techniques, the number of spectral peaks in the molecular mass window of 3–11 KDa was limited to a maximum of 50. Relative intensity of the smallest peak was >2% of the most abundant peak. Percent distribution of peaks and most abundant peaks were calculated based on signal of peaks within the active mass range. Within the fingerprint data module, global band matching was performed to mark multiple banding patterns. Dendrograms along with similarity matrices were generated using cluster analysis with Pearson correlations. Tree stability was assessed by bootstrap analysis with 1,000 iterations. Spectral fingerprint data was overlayed to compare the mass spectra generated for different strains of MAP and species of mycobacteria.

## Results

### Differentiating mycobacteria at the species level

**Figure 1:**
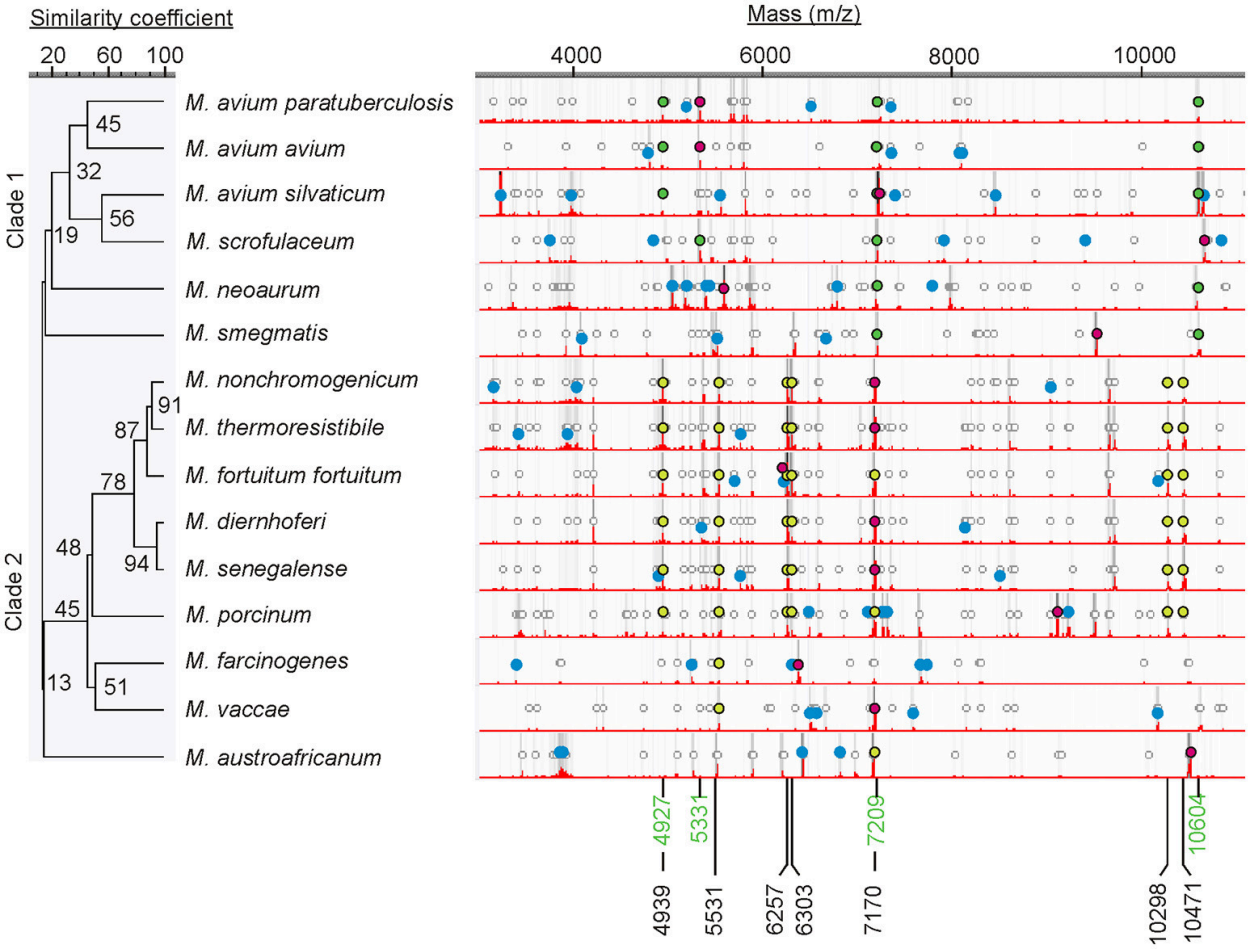
Dendrogram showing mass spectral similarities between different mycobacteria. Circular markings denote peaks that are >2% of the most abundant peak. Relative darkness of gray lines corresponds to relative intensity of the peaks. Spectra show most abundant peaks (red dots), peaks unique to each organism (blue dots), and finger prints common to each other within each clade (clade 1, green dots; clade 2, yellow dots). Molecular ions common to each clade are also marked (clade 1, green m/z; clade 2 black m/z). Mass spectrum for MAP reference strain ATCC 19698 is shown in this comparison.

MALDI-TOF analysis revealed distinct spectral profiles for each of the 15 different species of mycobacteria (Figure 1) that could be used to differentiate them from each other (Table 2). However, similarities in spectral profiles were observed between the members of *M. avium* complex and between all mycobacteria tested. Based on such similarities in peak profiles mycobacteria were grouped into two distinct clades (Figure 1). Clade 1 was dominated by members of *M. avium* complex, and clade 2 with mostly fast growing members of mycobacteria. Molecular ions 4,927, 5,331, 7,209, and 10,604 were common in spectra of most members of clade 1, whereas molecular ions 4,939, 5,531, 6,257, 6,303, 7,170, 10,298, and 10,471 were common to most in clade 2.

**Table 2:** Spectral characteristics unique to each species of *Mycobacterium* tested.

**Species**	**M/z (percent most abundant peak^b^)**
MAP^a^	5,332 (100), 5,691 (72), 10,618 (71), 5,828 (66), 5,785 (65), 7,236 (56), 10,598 (50), 4,929 (48)
*M. avium* subsp. *Avium*	5,329 (100), 4,799 (76), 8,100 (65), 7,348 (31), 8,083 (25)
*M. avium* subsp. *Silvaticum*	7,226 (100), 3,215 (77), 8,462 (27), 10,657 (27)
*M. scrofulaceum*	10,690 (100), 3,741 (30), 7,921 (28), 9,408 (23), 4,982 (23)
*M. neoaurum*	5,589 (100), 5,038 (54), 7,987 (46), 5,389 (45), 5,169 (40), 6,781 (31)
*M. smegmatis*	9,537 (100), 4,065 (33), 5,471 (28), 6,593 (24)
*M. nonchromogenicum*	7,186 (100), 4,005 (22), 9,052 (21), 3,142 (16)
*M. thermoresistibile*	7,187 (100), 5,760 (17), 3,920 (12), 3,396 (9)
*M. fortuitum* subsp. *Fortuitum*	6,256 (100), 10,279 (10), 5,497 (8), 6,240 (7)
*M. diernhoferi*	7,187 (100), 5,236 (12), 8,130 (9)
*M. senegalense*	7,187 (100), 5,403 (15), 8,510 (10), 4,866 (8)
*M. porcinum*	9,119 (100), 9,240 (42), 7,662 (38), 7,272 (37), 7,321 (34), 6,494 (33)
*M. farcinogenes*	6,379 (100), 7,680 (63), 5,095 (29), 7,746 (22), 6,363 (22), 3,380 (18)
*M. vaccae*	7,186 (100), 10,186 (41), 6,502 (31), 7,591 (18), 6,664 (15)
*M. austroafricanum*	10,529 (100), 6,423 (81), 10,512 (51), 3,863 (28), 3,878 (22), 6,815 (22)

a *Percent of most abundant peak values for MAP reference strain ATCC 19698 are shown*.

b *Most abundant peak for each species of Mycobacterium is also shown*.

### Differentiating strains of MAP

**Figure 2:**
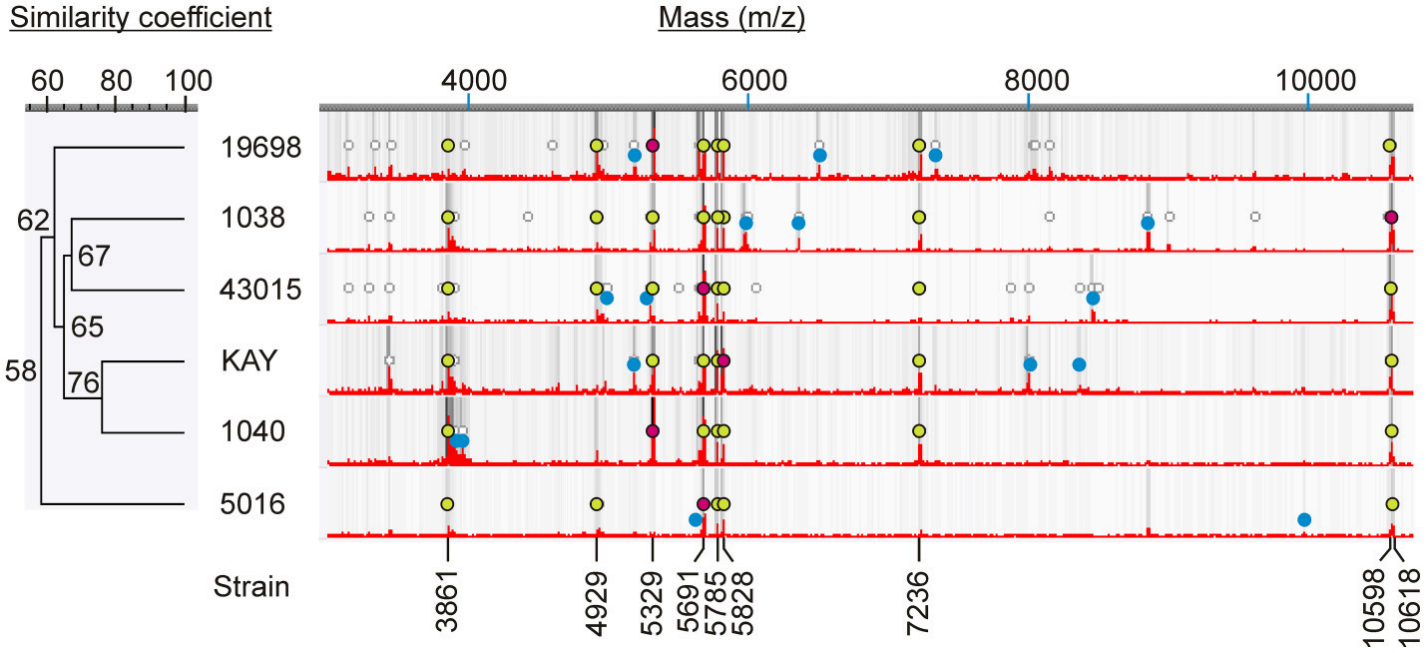
Mass spectral characteristics of MAP strains. Circular markings denote peaks that are >2% of the most abundant peak. Relative darkness of gray lines corresponds to relative intensity of the peaks. Mass spectra show most abundant peaks (red dots), peaks unique to each strain (blue dots) and peaks common to all (yellow dots).

Six different strains of MAP were analyzed by MALDI-TOF and found that all spectra contained several peaks (m/z 3,861, 4,929, 5,329, 5,691, 5,785, 5,828, 7,236, 10,598, and 10,618) in common (Figure 2), but also contained peaks that were unique to each strain (Table 3). Most intense peaks were also different for different strains of MAP. In addition, the dendrogram show more than 58% similarity in peak profiles between all MAP strains, whereas expression profiles of bovine strains NADC Kay and NADC 1040 showed most similarity of 76%. Furthermore, the spectral profile of human strain ATCC 43015 was 67% similar to that of the bovine strain NADC 1038. Spectral profiles of repetitive runs for strain ATCC 19698 on different days were essentially similar.

**Table 3:** Spectral characteristics unique to each MAP strain.

**Strain**	**M/z (percent most abundant peak^a^)**
ATCC19698	5,332 (100), 5,654 (68), 7,241 (56), 6,511 (37)
NADC 1038	10,616 (100), 5,984 (42), 6,368 (32), 9,018 (21)
ATCC 43015	5,691 (100), 5,308 (27), 8,474 (26), 4,960 (18)
NADC KAY	5,825 (100), 8,014 (59), 5,191 (52), 8,383 (35)
NADC 1040	5,329 (100), 3,902 (56), 3,882 (34), 3,961 (25)
NADC 5016	5,692 (100), 5,677 (37), 9,986 (26)

a *Most abundant peak for each MAP strain is also shown*.

### Similarity of mass spectral profiles of mycobacteria

**Figure 3:**
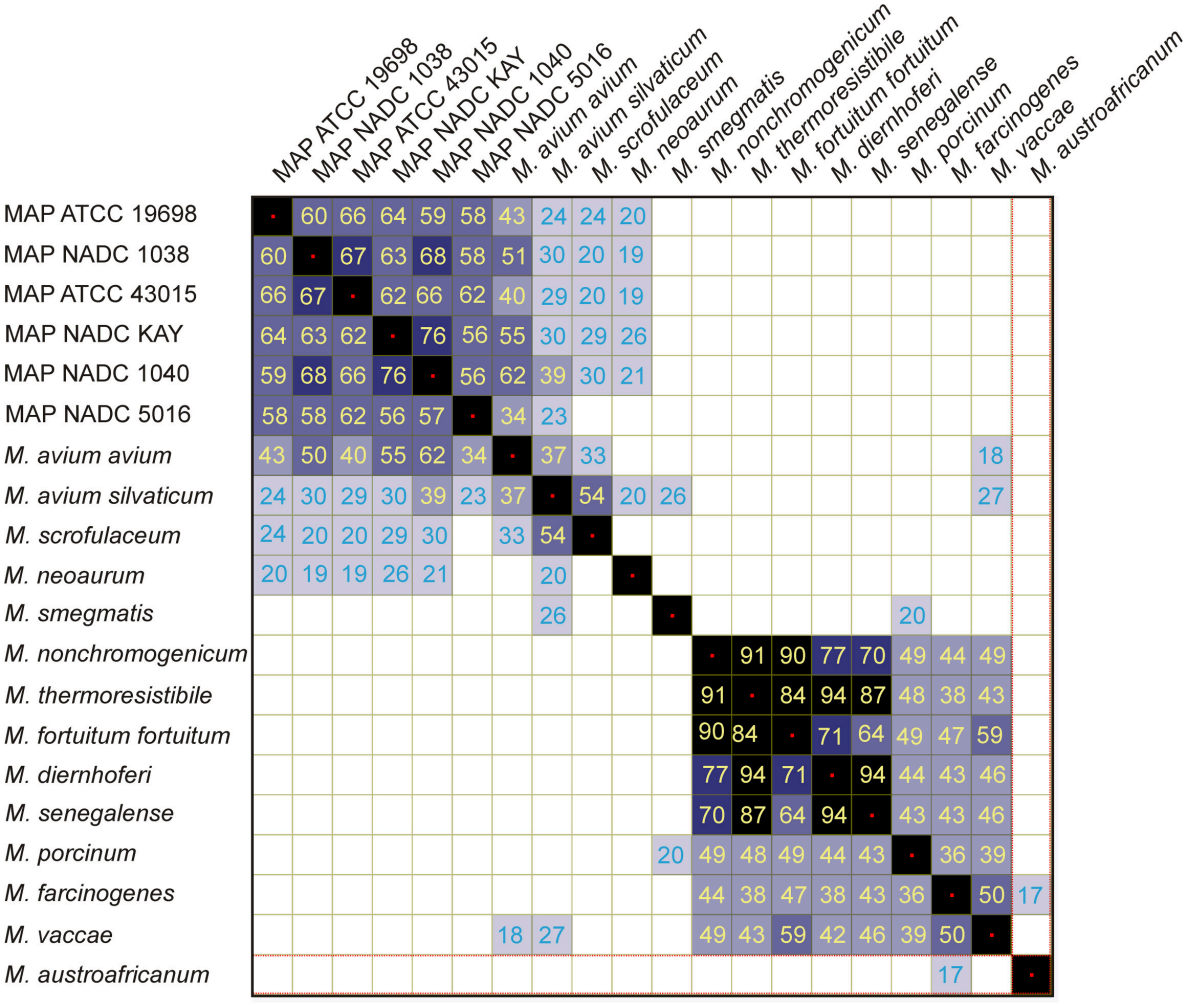
Similarity coefficient matrix for mycobacteria.

Cluster analysis with Pearson pair-wise comparisons of mass spectral profiles indicated two distinct clusters (Figure 3). Similarity in spectral profiles between MAP strains was as high as 76%, while the similarity for *M. avium* subsp. *avium* and MAP *strains* ranged between 34 and 62%. Members of clade 2 were highly related by the spectral profiles. For example, a 94% similarity in expression was observed between *M. diernhoferi* and *M. thermoresistibile* and between *M. diernhoferi* and *M. senegalense. M. neoaurum, M. smegmatis*, and *M. austroafricanum* were unrelated by cluster analysis.

## Discussion

It is essential to minimize the spread of MAP because of the significant economic losses to the US dairy industry and the association of this deadly pathogen with human autoimmune diseases. Confirming the causation requires tracking the pathogen to the source. Molecular typing methods for trace back studies are available that discriminate strains of MAP (Amonsin et al., 2004; Oakey et al., 2014), but molecular typing of large collection of strains is costly and time consuming. Molecular methods require extraction and purification of DNA, PCR amplification of the target sequences and followed by sequencing the targets. Alternatively, the inexpensive and rapid MALDI-TOF MS method (Samli and Ilki, 2016) described here uses protein profiles (Park et al., 2016; Penny et al., 2016) of pure cultures to type MAP strains and further differentiate them from other mycobacteria.

Extraction of proteins while disrupting cells by bead beating in TFA extraction solution resulted in clean and resolved spectral peaks in the mass window of 3–11 KDa range. Similar extraction procedures were used to obtain quality spectral profiles for enteric pathogens *E. coli* O157:H7 (Fagerquist et al., 2010) and *Campylobacter* (Mandrell et al., 2005; Penny et al., 2016). Likewise, lysates obtained by cell disruption using glass beads were used to obtain quality profiles for clinical isolates of *M. avium* and *M. tuberculosis* complex organisms (Machen et al., 2013). However, incomplete extractions or un-extracted whole cells spotted directly on MALDI plates resulted in dissimilar spectral profiles (Hettick et al., 2006; Pignone et al., 2006; Lotz et al., 2010; Lin et al., 2013). Thus, sample preparation techniques define the quality and composition of protein expression profiles.

We were able to differentiate MAP strains from 14 other mycobacterial species from bovine, human and environmental sources. In addition, mycobacterial species were differentiated from each other based on their unique spectral profiles (Table 2). Three to eight distinct protein biomarkers were used in distinguishing mycobacteria at the species level. Cluster analysis resulted in two distinct groups (Figure 3) each with common spectral signatures (Figure 1). It is remarkable that all the slow growing members of the avium complex had expressed protein profiles that are distinctly different from fast growing members of clade 2. Furthermore, the protein profiles are so distinct that taxonomically related avium complex group subspecies *paratuberculosis, avium*, and *silvaticum* can be distinguished from each other (Table 2, Figure 1).

A greater degree of similarity in protein expression profiles of fast growing mycobacteria was found. Although DNA-DNA hybridization studies indicated that *M. diernhoferi* of *M. parafortuitum* clade and *M. senegalense* of *M. fortuitum* clade are of distinctly separate species (Baess, 1982), a 94% similarity in protein profiles was observed between both organisms. Similarly, genetically distinct strains *M. nonchromogenicum* and *M. thermoresistibile* have similar mycolic acid production patterns (Minnikin et al., 1984) and protein expression profiles (91% similar).

It is remarkable that MALDI-TOF differentiated all six strains of MAP from each other by their unique protein expression profiles (Table 3, Figure 2). Three to four distinct protein biomarkers were chosen for each strain to aid in differentiating them from each other. Currently differentiation of genotypes of MAP may have been possible only with the time consuming MLSSR genotyping coupled with MIRU-VNTR typing (Douarre et al., 2011) or by mass spectral characterization of short sequence repeats (Ahlstrom et al., 2014). Although the findings reported here need further validation using a larger collection of MAP strains, protein expression profiling by MALDI-TOF MS may aid in rapid classification of MAP genotypes. Both MALDI-TOF and DNA based typing methods could be used complementary to each other in source tracking the pathogens.

Similarity matrix data generated using protein expression profiles of mycobacteria indicate two distinct clusters (Figure 3). It is noteworthy that there is not a greater degree of similarity (<76%) in protein expression between strains of MAP and thus they could be easily distinguished from each other. It is also remarkable that the MAP human strain is 62–66% similar to the other bovine isolates. In contrast, a higher degree of similarity (94%) was noted at species level for *M. diernhoferi, M. senegalense*, and *M. thermoresistibile*. Protein profile based typing would have been difficult with only a 6% differences in spectral profiles for these organisms. Nonetheless, they were differentiated by the presence of a few unique peaks (Figure 1, Table 2).

In conclusion, we analyzed lysates of cells by MALDI-TOF MS and obtained protein expression profiles for mycobacteria. MAP strains were differentiated from each other and from various species of mycobacteria. This rapid MALDI-TOF method can be used in classifying and source-tracking MAP strains in that the spectral profiles are vastly different between isolates.

## Author contributions

SR, LH, and CS equally contributed in designing and conducting the experiments. SR has written the manuscript and LH and CS contributed in preparation of the manuscript.

### Conflict of interest statement

The authors declare that the research was conducted in the absence of any commercial or financial relationships that could be construed as a potential conflict of interest.
